# The ‘can do, do do’ concept in COPD; quadrant interpretation, affiliation and tracking longitudinal changes

**DOI:** 10.1186/s12931-020-01375-3

**Published:** 2020-05-12

**Authors:** A. J. Alex van ’t Hul, E. H. Noortje Koolen, H. W. Jeroen van Hees, B. Bram van den Borst, M. A. Martijn Spruit

**Affiliations:** 1 Department of Pulmonary Diseases, Radboud University Medical Center, 6525 GA Nijmegen, The Netherlands; 2 Department of Research and Education, CIRO+, Centre of Expertise for Chronic Organ Failure, CIRO, 6085 NM Horn, The Netherlands; 3 Department of Respiratory Medicine, Maastricht University Medical Centre, NUTRIM School of Nutrition and Translational Research in Metabolism, 6229 HX Maastricht, The Netherlands; 4 REVAL-Rehabilitation Research Center, BIOMED-Biomedical Research Institute, Faculty of Rehabilitation Sciences, Hasselt University, 3590 BE Diepenbeek, Belgium

## Main text

We greatly appreciate the efforts made by Sievi and colleagues to verify our recent findings about the ‘can do, do do’ concept for patients with COPD and expand our understanding on the long-term dynamics of the quadrant affiliation [1, 2]. Since a personalized intervention to improve physical functioning for a patient with COPD may be derived from the quadrant affiliations, interventions should be congruent with the actual ‘can do, do do’ status. We feel challenged, however, to discuss this peer-reviewed paper publicly, with focus on the authors’ interpretation of our findings and two methodological issues.

## Lazy

Sievi and colleagues claim that we have described patients with COPD in the ‘can do, don’t do’ quadrant as lazy. This is further emphasized in the article’s title. However, we have never used the word ‘lazy’ in our paper and we never intended to even give the suggestion that these patients simply ‘don’t do’. That would be a too unidimensional approach. It is well documented that patients with COPD exhibit multiple physical, emotional and socials barriers and enablers to be engaged in physical activity [3]. As patients in the ‘can do, don’t do’ quadrant have a relatively preserved physical capacity, it is likely that the main determinant(s) of the low habitual physical activity needs to be found in the behavioral aspects. Therefore, a further analysis on all these aspects is required to customize an appropriate intervention for patients in the ‘can do, don’t do’ quadrant. This is far from simple and likely requires an interdisciplinary healthcare professional team approach.

## Reference values

To enable a head-to-head comparison between our findings and those of Sievi and colleagues, the cohort, test methodology, reference values and cut points to classify patients in the ‘can do, do do’ quadrants needs to be similar. In our study, we included COPD patients upon first referral to secondary pulmonary care, while the cohort of Sievi et al. comprised of patients already in secondary care. This may have led to the selection of a more severely impaired cohort in the Swiss study, which, compared to our study, is indeed reflected in a lower mean FEV_1_ (44 versus 56%pred), a lower median physical activity level (4421 versus 5112 steps/day), and a lower median 6-min walking distance (6MWD; 418 versus 440 m, respectively). Then it would not surprise if quadrant representation would differ between the studies, where we anticipated that Sievi’s study have proportionally more patients in the “can’t do” quadrants and less in the “can do” quadrants. However, the opposite is true. Sievi’s cohort had only 35% of patients in the “can’t do” quadrants versus 55% in our study, and as much as 65% in the “can do” versus 45% in our study. This discrepancy can probably be explained on the basis of 6MWD reference values. While we applied the Troosters’ reference values [4], Sievi and colleagues used those of Enright [5] of which we know that they significantly overestimate exercise capacity [6]. This has likely caused the shift in ‘can do’ – ‘can’t do’ proportions and precludes true comparison with our study. Considering this 6MWD reference is a key factor in the ‘can do, do do’ concept, we are curious to learn how this would alter the proportions and characteristics of the quadrants in Sievi’s cohort. Moreover, we anticipate that the graph in Figure 2 mistakenly puts the PA cut point at 75% as opposed to the proposed 70%.

## Interpreting longitudinal change

Studying the (in) stability of the ‘can do, do do’ quadrant affiliation longitudinally is challenging. Theoretically, each patient in any quadrant has four possibilities when assessed a second time in follow-up: 1) remain in the same quadrant, 2) improve or decline in terms of PA, 3) improve or decline in terms of PC, or 4) improve or decline in both PA and PC. Yet, while Sievi et al. rightfully argued that PA and PC are different constructs of physical functioning, they combined improving or declining in either PC or PA into “improvers” or “decliners”. This makes the interpretation of any quadrant change very difficult. Interpretation of change becomes even more complicated when more than two longitudinal assessments have taken place. This introduces a fifth category which they termed waverers, i.e. those who initially increased and later decreased (or vice versa) either or both PA and PC. Sievi et al. found no clear differences between remainders, improvers, decliners and waverers in explorative analyses. While the gradual loss of statistical power along with decreasing number of patients in the follow-up analyses is likely an explanatory factor, such an analysis should correct for events and circumstances affecting what patients ‘can do’ and ‘do do’. Factors that should be accounted for are: (1) events resulting in prompt deterioration, for instance acute exacerbations, (2) gradual decline, for instance as a results of pulmonary function impairment progression or worsening of other accompanying conditions, i.e. comorbidities, and/or, (3) interventions potentially improving what patients ‘can do’ and ‘do do’ such as rehabilitation which appeared from a recent study to be to highly unpredictable [7]. Moreover, it is important to acknowledge that clinically relevant improvements in physical functioning can occur without a change in quadrant affiliation [7]. Finally, would changes in quadrant allocation over time occur, then it does not in any way diminish the applicability of the ‘can do, do do’ concept to provide customized care to improve physical functioning.

It was difficult to understand from Sievi’s paper how decliners and improvers were exactly defined. Did any prespecified change across the borders of physical capacity (70% of the predicted 6MWD value) and/or physical activity (5000 steps/day) over time, result in a change in group affiliation, or was the minimal clinically important difference (MCID) applied? In the methods section, the authors mention the MCID’s for 6MWD and steps/ day. However, the concept of MCID does not consider the test-to-test variability, does not distinguish true change on a 6MWD from random measurement error, and is therefore improper to use on an individual level to detect any differences in exercise capacity [8]. Although the 6MWD is considered to be a reliable measure of exercise capacity, large limits of agreements are found between repeated measurements in the order of − 71 up to 148 m [9]. Following this reasoning, we are curious to know what would be left from the % of patients that change between quadrants over time if these values had been used.

## Data Availability

NA

## Abbreviations

PC: Physical capacity
PA: Physical activity
6MWD: 6-min walking distance
MCID: Minimal clinically important difference

## References

[1] Sievi NA, Brack T, Brutsche MH, Frey M, Irani S, Leuppi JD, Thurnheer R, Kohler M, Clarenbach CF (2020). “Can do, don’t do” are not the lazy ones: a longitudinal study on physical functioning in patients with COPD. Respir Res.

[2] Koolen EH, van Hees HW, van Lummel RC, Dekhuijzen R, Djamin RS, Spruit MA, van ‘t Hul AJ (2019). “Can do” versus “do do”: a novel concept to better understand physical functioning in patients with chronic obstructive pulmonary disease. J Clin Med.

[3] Gimeno-Santos E, Frei A, Steurer-Stey C, de Batlle J, Rabinovich RA, Raste Y, Hopkinson NS, Polkey MI, van Remoortel H, Troosters T, Kulich K, Karlsson N, Puhan MA, Garcia-Aymerich J, consortium PR (2014). Determinants and outcomes of physical activity in patients with COPD: a systematic review. Thorax.

[4] Troosters T, Gosselink R, Decramer M (1999). Six minute walking distance in healthy elderly subjects. Eur Respir J.

[5] Enright PL, Sherrill DL (1998). Reference equations for the six-minute walk in healthy adults. Am J Respir Crit Care Med.

[6] Andrianopoulos V, Holland AE, Singh SJ, Franssen FM, Pennings HJ, Michels AJ, Smeenk FW, Vogiatzis I, Wouters EF, Spruit MA (2015). Six-minute walk distance in patients with chronic obstructive pulmonary disease: which reference equations should we use?. Chron Respir Dis.

[7] Osadnik CR, Loeckx M, Louvaris Z, Demeyer H, Langer D, Rodrigues FM, Janssens W, Vogiatzis I, Troosters T (2018). The likelihood of improving physical activity after pulmonary rehabilitation is increased in patients with COPD who have better exercise tolerance. Int J Chron Obstruct Pulmon Dis.

[8] Dolmage TE, Hill K, Evans RA, Goldstein RS (2011). Has my patient responded? Interpreting clinical measurements such as the 6-minute-walk test. Am J Respir Crit Care Med.

[9] Singh SJ, Puhan MA, Andrianopoulos V, Hernandes NA, Mitchell KE, Hill CJ, Lee AL, Camillo CA, Troosters T, Spruit MA, Carlin BW, Wanger J, Pepin V, Saey D, Pitta F, Kaminsky DA, McCormack MC, MacIntyre N, Culver BH, Sciurba FC, Revill SM, Delafosse V, Holland AE (2014). An official systematic review of the European Respiratory Society/American Thoracic Society: measurement properties of field walking tests in chronic respiratory disease. Eur Respir J.

