# Correction to: The ‘can do, do do’ concept in COPD; quadrant interpretation, affiliation and tracking longitudinal changes

**DOI:** 10.1186/s12931-020-01397-x

**Published:** 2020-05-23

**Authors:** Alex J. van ’t Hul, Noortje H. Koolen, Jeroen W. van Hees, Bram van den Borst, Martijn A. Spruit

**Affiliations:** 1 Department of Pulmonary Diseases, Radboud University Medical Center, 6525 GA Nijmegen, The Netherlands; 2 Department of Research and Education, CIRO+, Centre of Expertise for Chronic Organ Failure, CIRO, 6085 NM Horn, The Netherlands; 3 Department of Respiratory Medicine, Maastricht University Medical Centre, NUTRIM School of Nutrition and Translational Research in Metabolism, 6229 HX Maastricht, The Netherlands; 4 REVAL-Rehabilitation Research Center, BIOMED-Biomedical Research Institute, Faculty of Rehabilitation Sciences, Hasselt University, 3590 BE Diepenbeek, Belgium

**Correction to: Respiratory Research (2020) 21:112**

**https://doi.org/10.1186/s12931-020-01375-3**

Following publication of the original article [1], the authors identified a mistake in the author names, as both forename and initials were stated.

Initially published author names:

A. J. Alex van ’t Hul, E. H. Noortje Koolen, H. W. Jeroen van Hees, B. Bram van den Borst and M. A. Martijn Spruit

Correct author names:

Alex J. van ‘t Hul, Noortje H. Koolen, Jeroen W. van Hees, Bram van den Borst, Martijn A. Spruit.

The original article has been corrected.
